# Micro-foundations of dynamic capabilities to facilitate university technology transfer

**DOI:** 10.1371/journal.pone.0283777

**Published:** 2023-03-30

**Authors:** Zhongxuan Ma, K. D. Augustijn, I. J. P. De Esch, B. A. G. Bossink

**Affiliations:** 1 Breakthrough Technology & Sustainable Innovation Research Group, Amsterdam Institute of Molecular and Life Sciences, Faculty of Science, Vrije Universiteit Amsterdam, Amsterdam, The Netherlands; 2 Division of Medicinal Chemistry, Amsterdam Institute of Molecular and Life Sciences, Faculty of Science, Vrije Universiteit Amsterdam, Amsterdam, The Netherlands; Universita degli Studi di Foggia, ITALY

## Abstract

Within the university-industry ecosystem, improvement and innovation of technology transfer involve implementing appropriate dynamic capabilities. To answer the question—What are the micro-foundations of dynamic capabilities in university technology transfer?—this study investigates in-depth organizational-level dynamic capabilities in transferring university-based knowledge to business and society. Two qualitative case studies were deployed at organizational entities at Vrije Universiteit Amsterdam: the Industry Alliance Office, and the Demonstrator Lab. These two organizations stimulate science- and business-oriented university technology transfer. In this context, the micro-foundations of the dynamic capabilities “sensing”, “seizing” and “reconfiguring” are identified and discussed. For “sensing”, which is the university’s ability to explore the opportunities in the ecosystem, the micro-foundations are “selecting internal competency” and “sensing external partners”. For “seizing”, which supports universities in managing complementarity with industry and society, micro-foundations include “resource co-allocation” and “collaborative business model”. The micro-foundations of “reconfiguring”, through which universities maintain evolutionary fitness in the innovation ecosystem, are “strategic renewal”, “establishing a university technology transfer-friendly environment”, and “asset orchestration”. This study provides researchers with a better understanding of how dynamic capabilities facilitate university technology transfer. Industrial practitioners and policymakers can consider the suggestions of the present study when pursuing collaboration with universities.

## 1 Introduction

Most universities have extended their activities to include a “third mission” besides education and research. This involves university technology transfer (UTT) that aims to increase the impact of academic research [1–5]. More specifically, UTT refers to transferring, converting, and commercializing university-based knowledge that has been developed at the universities to benefit universities, industry, and society [3, 6]. To gain higher UTT performance, universities must manage the wide variety and heterogeneity of tangible and intangible assets, including internal and external financial, commercial and human capital resources [4, 7, 8].

Recent academic interest highlighted the role of dynamic capabilities as a valuable strategic instrument for academic and industrial practitioners in UTT to understand how technology, organization, and strategy interrelate and interact in the collaborative university-industry innovation ecosystem [9–11]. The theory of dynamic capabilities emphasizes that universities should develop organizational capabilities to sense, seize, and reconfigure their strategic assets to improve the quality and prestige of their research, teaching, and public service [10, 12]. Optimizing dynamic capabilities would strongly support their role as orchestrator of the ecosystem and achieve the “third mission” [10].

To date, researchers have illustrated the role of responsible leadership and strategic management at the macro level of the university [9, 13, 14]. In contrast, few empirical studies have specifically studied the managerial routines in the UTT process. Concerning novel UTT models that are constantly emerging and evolving, best practices are likely to be hidden in the universities’ day-to-day management process, while others might be bundled with previously existing collaborative models. Moreover, one main obstacle revealed in a recent study is how to maintain R&D collaborations between universities and companies over a lengthy period [15, 16]. Merely focusing on the overall university management will not provide detailed insights into the dynamics of the management processes of UTT [12]. If appropriately managed, universities will function as vital creators, incubators, and connectors of university-enabled knowledge in terms of new treatments, scientific findings, and innovative inventions [10]. So far, however, the role of dynamic capabilities is underappreciated in university-industry collaborative (UIC) ecosystem research. Many previous studies are either incomplete or too general and need more substantiation [10, 12]. Overall, an important research gap is that few empirical studies have revealed the micro-foundations of dynamic capabilities in the UTT process and how these micro-foundations change with regard to the contextual dynamics.

To help to fill this gap, the present study is centered on the research question: *what are the micro-foundations of dynamic capabilities in university technology transfer*? This study aims to capture new empirical insights from the UTT process in practice while using the theoretical lens of dynamic capabilities. It uses a qualitative multiple case study as the research strategy and uses a deductive framework to match the empirical assets between the UTT phenomena and the selected theoretical lens [17, 18]. This research approach links multiple types of qualitative data with the established theoretical framework of dynamic capabilities. By extracting managerial patterns and routines that either stimulate or hinder UTT within the selected cases, the present study aims to identify the underlying mechanisms and their relationships, therefore contributing to the development of the chosen theory [17, 18].

It investigates the micro-foundations, or, specific origins, of the dynamic capabilities of university technology transfer by studying two UTT cases at Vrije Universiteit Amsterdam in Netherlands: the Industry Alliance Office (IAO) and the Demonstrator Lab (D-lab). IAO is an initiative from the Amsterdam University Medical Centers, with the aim to promote university technology transfer from a scientific perspective by spawning collaborative R&D projects. Demonstrator Lab (D-lab) is an incubating platform that transfers university-based knowledge into business by facilitating academic entrepreneurship. These cases are relevant since, on the one hand, they transfer university technologies towards R&D projects and startup companies, which represent the science and business-oriented approach of university technology transfer, respectively [3]. On the other hand, housing in the same university-industry ecosystem ensures institutional and environmental stability between these two cases, contributing to the analytical generalizability of the conclusions in the present study to comparable cases. For industrial practitioners, this article shows how business-oriented UTT practices are conducted, which provides a reference for them to join. For policymakers, this study provides insights into basic strategies and practices of UTT innovation that provide suggestions to them to establish relevant incentives.

## 2 Theory

### 2.1 From the resource-based view of the firm to dynamic capabilities

With a firm-specific perspective, the resource-based view (RBV) emphasizes the internal strategic resources and competencies that deliver superior competitive advantages to firms [19]. These “strategic resources” are defined as VRIN-resources, which means that these are Valuable, Rare, Inimitable, and Non-substitutable [20]. RBV proposes that a company can gain sustainable competitiveness if it can control endogenous VRIN resources [21, 22]. Apart from studying the deployment of endogenous strategic resources, investigating how organizations manipulate resources from outside of their organizational boundaries is equally important [22]. In the literature, RBV has received criticism for neglecting the contribution of the micro‐dynamics of resource development, procedures of value creation, capability renewal over time, and the relation between the business model and its contextual environment [23, 24]. In response to this concern, RBV was extended with theory about dynamic capabilities, which substantiates that organizations can “sense” internal and external demands, redeploy or “seize” VRIN resources, and transform or “reconfigure” management competencies to become more adaptable to the changes in the surrounding environment [25–27]. According to the previous literature, dynamic capabilities refer to firm’s managerial routines and procedures that enable these organizations to sense, seize, and reconfigure the strategic resources to match the environmental changes [22, 25]. These innovative capabilities are a series of managerial procedures that improve internal technological innovation capability by integrating strategic resources and reconstructing innovation processes in the environment [27]. Accordingly, dynamic capabilities can be regarded as the results of a firm’s conscious orientation and are embedded in innovative managerial routines.

Within the university-industry ecosystem, dynamic capabilities could positively catalyze and enhance the innovation ecosystem and stimulate the potential of universities [12]. The innovative procedures based on dynamic capabilities equip organizations with evolutionary fitness and promising innovation performance [28–30]. However, few studies focus on the micro-foundations of dynamic capabilities within university-industry collaborative (UIC) settings [12, 31], which may hinder the university and industry in managing and maintaining a sustainable UIC ecosystem.

### 2.2 Dynamic capabilities in university technology transfer

Recent literature has focused on the role of dynamic capabilities in the management of the UIC ecosystem since universities, like for-profit organizations, also need to face complexity on many fronts [9, 11, 12]. This includes integrating multi-stakeholder groups with conflicting goals, facing the uncertainty in governmental funding, and deploying digitally enhanced learning and teaching facilities on top of managing a difficult-to-forecast future, for example, as was recently underlined by the corona pandemic [10, 12]. Therefore, universities need to develop organizational capabilities to become more resilient, innovative, and competitive in a turbulent and uncertain environment. These organizational capabilities include, but are not limited to, continuously sensing the changes in the surrounding context, allocating both internal and external resources, orchestrating innovative assets, and improving evolutionary fitness and sustainability. Previous studies have outlined a critical role of dynamic capabilities for universities in aligning strategic assets in the UIC ecosystem to capture and maintain value in both entrepreneurship- and science-oriented UTT contexts [5, 6, 11, 32]. Recent literature has begun to examine the dynamic capabilities in university UTT; however, little attention has been paid to the micro-foundations, more specifically: to the exact origins of the dynamic capabilities “sensing”, “seizing” and “reconfiguring” that lie in the management routines of organizations [9, 11, 26].

#### 2.2.1 Sensing

Universities deploy sensing capabilities to discover and determine opportunities within the UIC ecosystem [11]. Researchers have identified influential resources that facilitate sensing ability, such as R&D technologies, financial support, and human resources [33]. The corresponding sensing capabilities, i.e., setting up routines to scan internal and external competencies, are crucial for universities to regulate innovation performance [34]. It is proposed that the outcomes of sensing capabilities are also influenced by the available strategies to pursue the potential opportunities [35]. Universities with strong sensing capabilities can better scan for off-campus partners and transfer university-generated knowledge into the UTT process.

#### 2.2.2 Seizing

Within the UIC ecosystem, seizing is defined as a set of managerial activities that help universities to create value based on previously sensed opportunities. These managerial routines include establishing technology transfer models for their intellectual properties and supporting technology commercialization. The process of technology commercialization requires universities to have efficient business models for UTT innovation [11]. During the seizing phase, resources can be allocated towards the perceived opportunities, helping the universities to build and maintain a sustainable partnership with industrial and governmental partners [12, 36]. The deployment of preemptive and initiative-taking capabilities allows for the close allocation of complementary assets on both sides of the university boundary. It helps maintain a sustainable ecosystem outside of campus [35].

#### 2.2.3 Reconfiguring

Reconfiguring refers to the managerial routines that universities use to sustainably revamp UTT processes and explore novel opportunities in university-industry collaboration by means of performing a system-orchestrating role in this collaboration [12, 37]. On the one hand, strategic assets, including financial, human, and social capital, differ among regional UIC ecosystems, even though they are all part of the same national innovation system [38–40]. On the other hand, the resources of an innovation system show uncertainty over time. Therefore, it is essential for universities to initiate reconfiguring capabilities for better value capturing and to improve their evolutionary fitness [41]. Technology transfer managers need to have a comprehensive understanding of how to orchestrate financial, labor, and technological assets in the market where the opportunities of UTT come from and where results of UTT are sold [42].

Above all, the micro-foundations of organizational dynamic capabilities are playing a significant role in catalyzing technological transfer and stimulating the potential of university-industry collaborations, towards which theoretical frameworks are waiting to be employed and explored in depth [10]. On the one hand, universities act as ecosystem orchestrators by deploying business-oriented UTT activities within the university-industry ecosystem. For example, some universities establish and manage an entrepreneurial science park as a business-based network for surrounding organizations (i.e., private companies, public sectors, and governmental agencies) to share and create value. On the other hand, universities as ecosystem participants that employ science-oriented activities to sense, seize, and reconfigure essential assets to improve internal R&D capabilities. Previous research highlights the important role of internal and external knowledge of universities in the regional ecosystem, but few studies investigate UTT routines and their changes over time to assess how to reinforce the UTT-related organizational dynamic capabilities [12, 43]. In response to this research gap, the present study reveals and tracks the managerial routines and their changes under the theoretical framework of dynamic capabilities. Accordingly, the findings provide suggestions to researchers and practitioners to measure, manage, and adjust relevant routines under a systematic theoretical mindset.

## 3 Material and methods

### 3.1 Selection of empirical setting

This study aims to capture new empirical insights from the UTT process in practice. According to previous studies, technological transfer and transition are presented and changed as organization- and application-specific routines that can be cumulated and measured along with strategic design, organization development, and project management [44–48]. The present study uses a deductive framework to match UTT routines and their changes with the micro-foundations of dynamic capabilities (see S1 Table for the found match between the micro-foundations of dynamic capabilities and the main UTT routines in the empirical research).

More specifically, the present study profiles two cases within Vrije Universiteit Amsterdam (VUA), thus making sure the consistency of the political and cultural contexts. Based on the different ways of UTT, academic entrepreneurship and collaborative science research are two representative models [3]. Regarding the UIC empirical settings in the current study, the first case, the Industry Alliance Office (IAO) of VUA is to promote transitional contract research of the neuroscience department in Amsterdam. The main purpose of the second case, the Demonstrator lab (D-lab) of VUA, is to act as the incubator for university-based spinoff companies. Therefore, the present study selects IAO and D-lab to indicate business- and science-oriented UTT (Table 1), making the analytical results relative and pertinent across the empirical cases and theoretical framework. This research strategy, combining within- and cross-case analysis, provides narrative descriptions of each case and analyzes the matching and different patterns between the cases [49, 50]. The interviewed informants are shown in S2 Table.

**Table 1:** Description of cases.

Case	Innovation category	Organizational goal	Initial focus on UTT innovation	The current focus on UTT innovation
Demonstrator lab (D-lab)	Business-oriented: academic entrepreneurship [3, 6]	D-lab is a successful university incubator that aligns the supporting resources of the Amsterdam Business Ecosystem towards entrepreneurial ideas from *knowledge institutes* in the city of Amsterdam.	Facilitating knowledge commercialization	Balancing knowledge creation and commercialization
Industry Alliance Office (IAO)	Science-oriented: collaborative research [3, 39]	IAO is a "one-stop" resource integrator of (bio)pharmaceutical companies worldwide and scientific researchers in Amsterdam Neuroscience, to speed up translational research.	Facilitating knowledge spillover	Facilitating the mutual promotion between knowledge creation and spillover

### 3.2 Data collection

To establish a solid qualitative empirical dataset, the present study collected a wide range of primary and secondary data, including semi-structured interviews, textual and visual data. The key informants of both cases include project coordinators, business developers, Chief Executive Officers (CEOs), Chief Scientific Officers (CSOs), founders of the startup companies, principal investigators (PIs), Ph.D. researchers, and other stakeholders who play important roles in the UTT process. For D-lab, this study initiated twelve semi-structured interviews and four informal interviews; for IAO, it initiated seven semi-structured interviews and one informal interview. The questions were semi-structured in advance (see S3 Table) and each interview lasted thirty minutes on average. For data triangulation purposes, one hundred and eighty-three secondary data sources were collected, including fifty publications, twenty-six video presentations and interviews, twenty-four descriptions on partner websites, and eighty-three news events. All data sources are presented in Table 2.

**Table 2:** Data sources of each case study.

Data sources	Case study		Total number
	Demonstrator lab	Industry Alliance Office	
Interviews			
Semi-structured interviews	12	7	19
Informal interviews	4	1	5
Archival data			
Video presentations and interviews	20	6	26
Publications	15	35	50
Descriptions on partner websites	17	7	24
News of events on websites	43	40	83

### 3.3 Data analysis

The present study employs a routine-focused research method to interview participants in the two cases about key routines and practices under a retrospective perspective, which implies the micro-foundations of dynamic capabilities and their changes. This study adopts the Gioia methodology to inductively interpret and code primary and secondary data in three stages [51], including opening-, second-, and theoretical- coding stages. In the opening coding stage, it established a list of first-order concepts based on informant and in vivo terms that were collected. These first-order concepts were formulated to describe the common practices from both cases, and to accurately represent the concepts used by key informants [52].

The second coding stage analyzes the similarities and differences between the first-order concepts and generalizes them to “second-order concepts”, representing the micro-foundations of each dynamic capability, by using a cross-case replication logic. The benefit of cross-case replication logic is that it helps to determine replicable concepts when these concepts are supported by multiple data sources in two or more cases [18]. Afterward, the relationship between the second-order concepts and related quotations was rechecked to improve the internal validity of the present study.

Thirdly, this study establishes a theoretical coding round to fine-tune the second-order concepts and aggregate them into the theoretical framework of dynamic capabilities. This data structuration stage was performed by selecting and aggregating second-order concepts according to their relationship with sensing, seizing, or reconfiguring. At the same time, it was examined whether there were nascent concepts in second-order concepts. Therefore, the study reduced the replicated empirical themes and integrated representative terms into a theoretical hierarchy of concepts. This study fine-tunes the definitive version of the data structure that formulates qualitative data from empirical settings to a dynamic capabilities framework inductively. S1 Table schedules the found match in these three stages between the micro-foundations of dynamic capabilities and the main UTT routines in the empirical research, and which will be narrated in the Results section.

### 3.4 Validity and reliability

Credibility and replicability of the results of this qualitative research need to be assured. At the same time, internal validity, which refers to the degree of consistency between the way key informants use a particular concept and how researchers describe and interpret that concept, also is an important quality criterion for this research [51, 53]. Finally, it is important to look for common insights for both cases, which indicate analytical validity, which means that these insights may also apply to other, comparable cases. This paper considers the following concerns to improve credibility, replicability and validity. 1) The present study follows a traceable research design, thus developing a clear case-study database, consisting of all empirical data that was gathered. 2) The raw interview data was collected based on a pre-structured outline wherein all the questions are related to the used theory. 3) The dynamic capabilities were tracked at the organizational level in a two-case study design, which simultaneously uncovers the detailed process of each specific UTT model, and of similar empirical patterns across these two cases. 4) The two-case study design, covering a business- and a science-oriented university-industry collaboration, and searches for and identifies similar empirical patterns, opting for finding insights with analytical validity for other, comparable UTT cases.

### 3.5 Ethic statement and participant consent

In the Netherlands, ethics review is only required by the Medical Research Involving Human Subjects Act (WMO) for (medical) research involving humans or animal models. Because this study is non-medical, it is exempt from ethics review. The research methodology of the present study is discussed with, and with the consent of all the participants, including research design, data collection, and data coding. Finally, this study does not involve vulnerable groups or confidential data.

## 4 Results

### 4.1 Sensing

#### 4.1.1 Industry alliance office

The Industry alliance office (IAO) has built up a robust R&D-based network for the Amsterdam Neuroscience Department by attending various “partnering conferences”, where IAO works in a focused and systematic way to meet as many relevant practitioners as possible. By doing so, business development managers reach out to the potential partners in the surrounding context and allocate and match the value-creation process between science and practice. One business developer of IAO used these conferences to meet with both parties, stating:

*“We go to academic conferences each year where we meet the scientists. … Some of those conferences have social events which is a good way for us to connect with industry.”*

Notably, IAO has formed a business development team where each member has accomplished a Ph.D. in biomedical or neurological science and is trained to master the skills of a business developer. Every business developer meets with about twenty principal VUA investigators regularly to catch up with their research updates, which helps IAO to match the right biotech company with the right scientific group. One business developer explained that:

*“There are lots of biotech companies that are initially led by scientists. It is important for you to have a double mind at this moment. They appreciate that you understand the content of what you are talking about.”*

#### 4.1.2 Demonstrator lab

Ten years ago, the Demonstrator lab was conceived as an “open venue” where academic entrepreneurs could help each other by being complementary, and share their personal networks, scientific information, and entrepreneurial thoughts. Even today, this strategy still shows its advantages for scientific researchers. One principal investigator said:

*“Over the years, I have always struggled with how we can commercialize the techniques that we use for fundamental science. … It was D-lab who encouraged me to think more and taught me how to learn more.”*

Besides incubating entrepreneurship among scientific researchers, Demonstrator lab also participates in M.Sc. and Ph.D. programs and sets up practice-oriented education. For example, Demonstrator lab collaborates with the VUA division of Science, Business & Innovation to provide both entrepreneurship-based education and internships to facilitate knowledge valorization for students [54]. The previous director of Demonstrator lab described that:

*“Initially, what I was thinking is to start a laboratory that could help researchers to bring their research to market. … Now we also set up courses and internships for Science, Business & Innovation students to help them to valorize the knowledge.”*

### 4.2 Seizing

#### 4.2.1 Industry alliance office

Industry alliance office has built up an appropriate business model for collaboration following a strategic procedure called the “four-step process”. Firstly, IAO formulates the needs of the external company in specific questions and queries. Subsequently, IAO evaluates whether VUA’s expertise or assets can meet these needs. Afterward, IAO matches complementary resources, and shapes a business model via negotiations, which are the third and fourth steps. During this stage, IAO works as an intermediary to manage conflicts between academia and industry. The Chief Science Officer at IAO described:

*“We have this strategy and standard contract templates. In addition, we hire professionals like lawyers to do the contract manufacturing.”*

Within the past ten years, IAO has mapped all scientific teams of the Amsterdam Neuroscience Department in one R&D pipeline, which enhances their ability to transform scientific knowledge into effective treatments in neurology and psychiatry. As the Chief Science Officer of IAO detailed:

*“What my team continuously does is to also look in the outside world to see if they develop drugs that align with the internal R&D pipeline here in the institutes.”*

#### 4.2.2 Demonstrator lab

Demonstrator lab provides early-phase academic entrepreneurs with personalized solutions that maximize the surrounding resources to offer all-around support, including education, advise, finance, infrastructure, and networking possibilities. Once involved, participants are facilitated with both lab and office spaces (for a total of 580 sqm) in place to launch the ideas. Demonstrator lab also provides academic entrepreneurs with no-string-attached “seed funding”, which is vital for early-phase academic entrepreneurship. By doing so, companies that are truly capable will exert their potential strength in such an entrepreneurially friendly environment. The initiator of D-lab mentioned that:

*“We do not have a complete business plan for everyone, but our ‘strategy’ is we are always in the way of operating. … We have a toolbox in hand to help different entrepreneurs based on their needs.”*

### 4.3 Reconfiguring

#### 4.3.1 Industry alliance office

The IAO set up a group of procedures, called “back-office support”, to deploy a dedicated and pragmatic contracting process between the business developers and principal investigators, making sure that every administrative and financial question will be addressed effectively. To track the collaboration and protect the data, IAO has built up a custom-made management system that safely collects, stores, and updates the data for ongoing projects. At the end of each collaboration, IAO encourages practitioners to build follow-up collaborations where the role of IAO will decrease and the role of practitioners themselves will increase. As a principal investigator from Amsterdam University Medical Center recounted:

*“You need to have, like, this long-term collaboration, and you have to be able to show that you really can deliver on what you promise. And at some moment, it is the trust that allows you to move into that interactive phase. That is important. … The IAO helps along the way to cover all the above issues.”*

#### 4.3.2 Demonstrator lab

Considering that there are so many startup projects, Demonstrator lab plans to evaluate itself under a more central perspective and set up more initiatives for knowledge valorization. On the one hand, Demonstrator lab continuously provides more entrepreneurship- and valorization-based courses for undergraduate, M.Sc., and Ph.D. researchers. On the other hand, Demonstrator lab is also working on exploring international sponsors to provide access for academic entrepreneurs with a cross-boundary perspective. For example, “*D-Lab has been officially part of the European Business Angels Network (EBAN) since April 2021*” (D-lab, archive data). Besides, Demonstrator lab works closely with other incubators to orchestrate entrepreneurial culture within the university such as the “VUA startup hub”. A manager of “VUA startup hub” said that:

*“As you can see, the Demonstrator lab is also involved in the Startup Hub. So, we will work together and combine our resources to better support our spinoff companies.”*

## 5 Discussion

**Fig 1:**
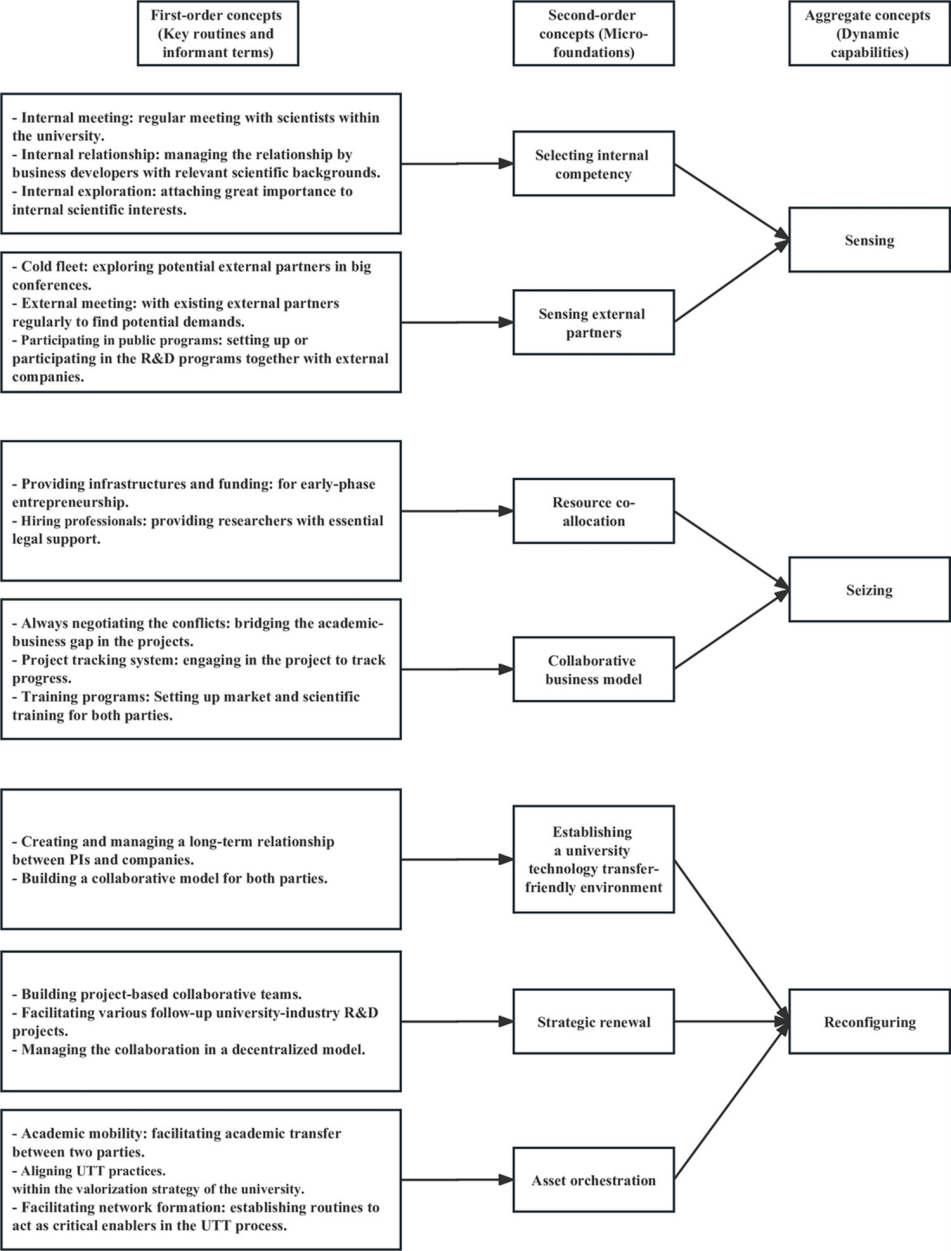
Structure of the empirical data.

The present study highlights that by applying dynamic capabilities-related micro-foundations and organizational routines, universities can specify the appropriate opportunities to commercialize their knowledge (Fig 1). The following Discussion section elaborates on the insights gained in the studied cases, while also citing literature that supports these findings.

### 5.1 Sensing

Sensing refers to universities’ organizational capabilities that strategically identify and filter the opportunities from the environment [10, 33]. Those opportunities come from the screening of the internal and external environment, which provides the cornerstone for resource allocation. Both cases establish unique procedures to explore the opportunities in the surrounding context.

#### 5.1.1 Selecting internal competency

Having a clear self-cognition of internal competency provides a solid foundation for UTT activities. Both IAO and D-lab have adopted this strategy by deploying various managerial activities for internal exploration. Especially for IAO, establishing and updating the internal R&D pipeline plays a vital role in sensing the internal competency. D-lab enhances its endogenous awareness of the entrepreneurial environment within VUA by orchestrating an entrepreneurship-based environment among the researchers. It is proposed that organizational performance is highly related to endogenous choice, towards which internal cognition provides the preconditions [55]. These dynamic routines initiated by IAO and D-lab also inspire scientists’ recognition of opportunities to transfer knowledge from their labs to the market [56].

#### 5.1.2 Sensing external partners

With the aim to explore external opportunities, it is important for universities to reach out to external partners and find a way to co-create value with them. Both IAO and D-lab have made significant efforts to attract potential partners via different methods, including retrieving European Union Calls for research proposals, attending collaborative or entrepreneurial conferences, and purposeful reaching out to the targeted authorities. Besides, both IAO and D-lab apply procedures that are related to sensing capabilities, not just at the initial stage of university technology transfer, but at every stage in the collaboration life cycle. It is proposed that deploying dynamic capabilities does not follow a linear trajectory, but an interactive and iterative pattern [57]. Accordingly, this study suggests practitioners follow a dynamic requirements-oriented model rather than a static pattern-oriented one when setting sensing capabilities in practice.

### 5.2 Seizing

Seizing refers to the organizational capabilities that can be used to mobilize resources so that the organization captures and creates value from previously discovered opportunities [25]. Both cases in the present study have routines to manage the interface between university and environment and engage in UTT activities to seize the previously sensed opportunities. These seizing routines that ensure both quality and quantity are of significant importance for UTT innovation [15].

#### 5.2.1 Resource co-allocation

Resource co-allocation together with external partners, including balanced assessment and efficient arrangement of aggregated resources, is a key element to increase the effectiveness of university’s UTT in terms of R&D management, technology adoption, and UTT innovation performance [3, 58, 59]. Specifically, IAO always engages in strategy formulation and resource fluidity along with its key stakeholders, while D-lab provides early-phase academic entrepreneurs with objective advisory support. Based on the empirical results, the present study indicates that setting up a common mission is an antecedent toward resource co-allocation, which equips universities with high-quality competencies to gain unique and non-interchangeable advantages [11]. As there are uncertainties in early-phase entrepreneurship and collaborative R&D, the spotlight can be shed more on how to establish the common mission and manage resource allocation for university technology transfer [60].

#### 5.2.2 Collaborative business model

Stakeholders in university-industry collaborations shape and manage collaborative business models that demonstrate how universities and industry create common value. To improve innovation performance, participants may need to leave their previously existing autonomous models and develop new tailor-made ones based on the collaborative value chain and targeted market [61]. Within the collaboration, it appears to be increasingly important for coordinators to manage and eliminate conflicts in technical, economical, and social dimensions during the process [3]. Regarding the cases, IAO invests in creating suitable business models for the collaboration where they function as a participant to manage conflicts in various scenarios, while D-lab acts as a resourceful advisor.

### 5.3 Reconfiguring

Reconfiguring indicates managerial routines that enable universities to renew and match their resources and competencies sustainably, therefore enhancing the evolutionary fitness in the regional economy [12, 62]. By employing iterative and stepwise managerial approaches, the two cases both strengthen the evolutionary fitness of the university and improve sensing and seizing capabilities in the meantime.

#### 5.3.1 Strategic renewal

To foster the long-term goal, “strategic renewal” refers to new managerial actions and processes that update and refresh organizational characteristics [58]. Every year, the directors of IAO and D-lab review their developmental trajectory and make updates. For universities, this would mean the need to manage the knowledge asymmetries and subsequent uncertainties, such as identifying, filtering, and interpreting information about technologies and market demands [33]. Universities often need to deploy managerial routines that reflect the flexibility of organizational structure and consistency of strategic objectives [63]. Their strategic renewal contributes to their evolutionary fitness, which support universities’ positions as central orchestrators in the university-industry ecosystem [31, 62–65].

#### 5.3.2 Establishing a university technology transfer-friendly environment

Building a UTT-friendly environment refines the collaborative model from organization-oriented to personalization-oriented, therefore equipping UTT activities with the ability to operate and evolve on their own [3, 66]. The IAO-supported R&D collaborations and D-lab-enabled entrepreneurship both show sustainable innovativeness, which benefits from the decentralized collaborative models they established. For both academic and entrepreneurial researchers, these organizational routines create a UTT-friendly environment where they spontaneously share knowledge with society beyond the walls of the university [67–69]. Accordingly, the present study suggests that decentralized UTT-friendly models help universities to avoid managerial conflicts due to over-engagement, and the outcomes tend to meet the concerns of both sides of the table.

#### 5.3.3 Asset orchestration

Based on the joint prosperity and common mission, asset orchestration refers to formal and informal consensus between university and industry [33, 70], which reinforces the co-evolutionary relationship between surrounding context and inter-organizational cooperation. In the present case study, IAO and D-lab continuously set up procedures to align their strategies with the valorization strategy of the host university, and operate as key enablers in the knowledge transfer chain. The above-mentioned managerial routines invigorate universities with the driving force to obtain sustainable development together with partnering companies [62, 71]. Under the perspective of dynamic capabilities, the evolutionary fitness of a university is shaped by and influences the surrounding innovation ecosystem [72]. New “synapses” are continuously generated, in which “neurotransmitters” of UTT will be released and transmitted. Therefore, this study also proposes that the capabilities of “sensing” and “seizing” are latent in and stimulated by the capabilities of “reconfiguring”.

## 6 Conclusions and implications

### 6.1 Conclusions

The present study aims to provide deeper insights into the micro-foundations of dynamic capabilities in the university-industry collaborative (UIC) context by employing an in-depth empirical study of science- and business-oriented university technology transfer (UTT) cases. Regarding the dynamic capability “sensing”, this study indicates that universities need to develop two micro-foundations that contribute to identifying and filtering opportunities from the surrounding environment. These are “selecting internal competency” and “sensing external partners”. Second, regarding the dynamic capability “seizing” this study finds that universities need to allocate complementarity with industrial companies and create value from previously sensed opportunities. The related micro-foundations include “resource co-allocation” and “collaborative business model”. Third, universities need the dynamic capability “reconfiguring” to enhance the sustainability of their current routines. The associated micro-foundations include “strategic renewal”, “establishing a university technology transfer-friendly environment”, and “asset orchestration”. The research findings show not only that and how dynamic capabilities carry weight; they additionally show which specific micro-foundations are active within these dynamic capabilities.

### 6.2 Implications

The present study deploys dynamic capabilities as the theory to study how to facilitate university technology transfer. The majority of scientific dynamic capabilities-based studies have focused on innovation scenarios of for-profit organizations, while so far few studies have been reported to deploy dynamic capabilities in university-industry collaborations, wherein not-for-profit universities are active [11, 31, 64, 71, 73]. The present study responds to the call to research the micro-foundations of dynamic capabilities in the UTT process, as previous studies mainly focus on the overall strategic management of universities [9, 12]. Moreover, the present study reveals micro-foundations and the changes of dynamic capabilities at the university level. In this context, the micro-foundations of the dynamic capabilities “sensing”, “seizing” and “reconfiguring” that can be measured and are operational in terms of routines in practice and research, are identified (see S1 Table), described and explained (see Results and Discussion sections of this research). The overview of the micro-foundations of dynamic capabilities and related organizational routines, which is based on an in-depth study of two complementary cases, yields insights that possess analytical validity; which means that these micro-foundations and related organizational routines can also play a role in UTT processes in other, similar cases.

Regarding practical implications, the present study provides practitioners and policymakers with an overview of the suggested organizational dynamic capabilities discovered in the empirical settings. By referring to these micro-foundations and related organizational routines, managers are more likely to better stimulate and manage the societal impact of university-based knowledge. For example, the present study suggests that a decentralized business model can offer both industrial and academic practitioners the means to orchestrate the integrated resources, with the aim to facilitate university technology transfer. For policymakers, our study suggests them to consider incentives and policies to arouse the development of novel innovation ecosystems around universities and gain more input from both researchers and practitioners.

## Supporting information

S1 Table Summary of the micro-foundations of dynamic capabilities and the main routines and changes in routines found in the study. (DOCX)

S2 Table Overview of informants. (DOCX)

S3 Table Interview questions. (DOCX)
