# Supplementary material for: Micro-foundations of dynamic capabilities to facilitate university technology transfer
Source: PLoS One. 2023 Mar 30;18(3):e0283777. doi: 10.1371/journal.pone.0283777 (PMC10062569; doi:10.1371/journal.pone.0283777)
Supplement: S1 Table — (DOCX) [file pone.0283777.s001.docx]

# Supporting information

**Table S1 - Summary of the micro-foundations of dynamic capabilities and the main routines and changes in routines found in the study**

| **Second-order concepts** | **First-order concepts** | **Main routines and the changes found in the study** |
| --- | --- | --- |
| **Micro-foundations** | **Replicated routines** |  |
| **Sensing** | | |
| **Selecting internal competency** | **Internal meeting** | 1. Arranging regular meeting with scientists about their research updates (IAO, D-lab).  2. Arranging regular entrepreneurial workshops for researchers (D-lab).  3. Recruiting business developers with scientific backgrounds (IAO). |
|  | **Internal relationship** |  |
|  | **Internal exploration** |  |
| **Sensing external partners** | **Cold fleet** | 1. Exploring potential external partners by emailing, calling, and then meeting in big conferences (IAO, D-lab).  2. Discussing and finding potential demands with external partners (IAO, D-lab).  3. Exploring opportunities from policy trends and open calls (IAO, D-lab). |
|  | **External meeting** |  |
|  | **Participating in public programs** |  |
| **Seizing** | | |
| **Resource co-allocation** | **Providing infrastructures and funding** | 1. Providing ‘seed funding’ for early-phase academic entrepreneurship (D-lab).  2. Providing necessary equipment for collaborative R&D projects (IAO).  3. Introducing analysist for academic entrepreneurs to solve IP-related issues (D-lab).  4. Hiring lawyers for researchers with professional legal supports (IAO).  5. Solving academic-business gaps by negotiating (IAO).  6. Providing entrepreneurial researchers with workshops to solve academic-business gaps (D-lab).  7. Engaging in the project to track R&D progress (IAO).  8. Establishing regular chats with researchers to follow the entrepreneurial process and provide assistance (D-lab).  9. Introducing researchers to market training programs (IAO).  10. Providing researchers with entrepreneurial training programs (D-lab). |
|  | **Hiring professionals** |  |
| **Collaborative business model** | **Always negotiating the conflicts** |  |
|  | **Project tracking system** |  |
|  | **Training programs** |  |
| **Reconfiguring** |  |  |
| **Establishing a UTT-friendly environment** | **Creating and managing a long-term relationship** | 1. Organizing seminars between academic researchers and industrial practitioners (IAO, D-lab).  2. Meeting with partners regularly after the projects (IAO).  3. Inviting representatives of partnering organizations into entrepreneurial workshops (D-lab).  4. Organize brainstorming to revisit collaborative projects (IAO).  5. Display of collaborative projects on the website (D-lab).  6. Participation in academic conferences to promote the results of cooperation (D-lab).  7. Always building a novel project team before the collaboration (IAO).  8. Helping researchers build up their entrepreneurial teams (D-lab).  9. Helping both parties find follow-up collaborative R&D opportunities (IAO).  10. Establishing fund to incubate potential collaborative projects (D-lab).  11. Setting up a flat structure during university-industry collaborative projects (IAO).  12. Using the network of entrepreneurial researchers to find new market opportunities and partners (D-lab).  13. Helping initiate science park on campus (D-lab).  14. Facilitating internships and visits to companies (IAO).  15. Aligning various R&D projects within the valorization strategy of the university. (IAO).  16. Aligning different entrepreneurial missions within the valorization strategy of the university (D-lab).  17. Acting as key enablers in the network-building process (IAO).  18. Participating in developing university's knowledge-valorization plan for the future (D-lab). |
|  | **Building a collaborative models for both parties** |  |
| **Strategic renewal** | **Building project-based collaborative teams** |  |
|  | **Facilitating various follow-up university-industry R&D projects** |  |
|  | **Managing the collaboration in a decentralized model** |  |
| **Asset orchestration** | **Academic mobility** |  |
|  | **Aligning UTT practices within the valorization strategy of the university** |  |
|  | **Facilitating network formation** |  |
