# Supplementary material for: Micro-foundations of dynamic capabilities to facilitate university technology transfer
Source: PLoS One. 2023 Mar 30;18(3):e0283777. doi: 10.1371/journal.pone.0283777 (PMC10062569; doi:10.1371/journal.pone.0283777)
Supplement: S2 Table — (DOCX) [file pone.0283777.s002.docx]

# Supporting information


**Table S2 - Overview of informants**

| **Serial Number** | **Date** | **Interviewee** | **Type** | **Location** |
| --- | --- | --- | --- | --- |
| 1 | 01/2022 | Director of D-lab | Open interview | Online (Zoom) |
| 2 | 11/2021 | Director of D-lab | Open interview | Online (Zoom) |
| 3 | 09/2021 | CSO of IAO | Open interview | Online (Zoom) |
| 4 | 09/2021 | Business developer of IAO | Semi-structure interview | Online (Zoom) |
| 5 | 09/2021 | Contract manager of IAO | Semi-structure interview | Online (Zoom) |
| 6 | 10/2021 | Business developer of IAO | Semi-structure interview | Online (On-site) |
| 7 | 11/2021 | Principal investigator of IAO | Semi-structure interview | Online (On-site) |
| 8 | 11/2021 | Principal investigator of IAO | Semi-structure interview | Online (Zoom) |
| 9 | 11/2021 | Principal investigator of IAO | Semi-structure interview | Online (Zoom) |
| 10 | 11/2021 | Ph.D. researcher of IAO | Semi-structure interview | Online (WhatsApp) |
| 11 | 10/2021 | Principal investigator in D-lab | Open interview | Online (Zoom) |
| 12 | 10/2021 | Principal investigator in D-lab | Open interview | Online (WeChat) |
| 13 | 10/2021 | Previous director of D-lab | Semi-structure interview | Online (On-site) |
| 14 | 10/2021 | Business developer in VUA | Semi-structure interview | Online (Zoom) |
| 15 | 11/2021 | Entrepreneur in D-lab | Semi-structure interview | Online (On-site) |
| 16 | 11/2021 | Entrepreneur in D-lab | Semi-structure interview | Online (On-site) |
| 17 | 11/2021 | Entrepreneur in D-lab | Semi-structure interview | Online (On-site) |
| 18 | 12/2021 | Entrepreneur in D-lab | Semi-structure interview | Online (Zoom) |
| 19 | 12/2021 | Entrepreneur in D-lab | Semi-structure interview | Online (Zoom) |
| 20 | 02/2022 | Entrepreneur in D-lab | Semi-structure interview | Online (Zoom) |
| 21 | 01/2022 | Principal investigator in D-lab | Semi-structure interview | Online (Zoom) |
| 22 | 02/2022 | Principal investigator in D-lab | Semi-structure interview | Online (Zoom) |
| 23 | 03/2022 | Principal investigator in D-lab | Semi-structure interview | Online (Zoom) |
| 24 | 04/2022 | Principal investigator in D-lab | Semi-structure interview | Online (Zoom) |
