# Supplementary material for: Micro-foundations of dynamic capabilities to facilitate university technology transfer
Source: PLoS One. 2023 Mar 30;18(3):e0283777. doi: 10.1371/journal.pone.0283777 (PMC10062569; doi:10.1371/journal.pone.0283777)
Supplement: S3 Table — (DOCX) [file pone.0283777.s003.docx]

# Supporting information

**Table S3 - Interview questions**

| **Interview types and theoretical dimensions** | | **Interview Questions** |
| --- | --- | --- |
| **Open interview** |  | - Would you like to introduce yourself and the innovation project?  - Would you like to generally introduce how you undertake the innovation project at our university? |
| **Semi-structured interview** | **Sensing** | D-lab:  - When did you (or your team) decide to bring the innovation project to the market?  - Who or what lead you (your team) to make this decision?  - Before making the decision, what did your team (D-lab) do?  IAO:  - When did you (or your team) decide to study the innovation project with external companies?  - Who or what lead you (your team) to make this decision?  - Before making the decision, what did your team (IAO) do? |
|  | **Seizing** | D-lab:  - What did your research team do after making the decision?  - What kind of activities has D-lab initiated to help researchers promote academic entrepreneurship?  - Did you encounter any difficulties in the process of academic entrepreneurship and how did you overcome these difficulties with the help of D-lab?  - How did partnership management influence the development of the growth of your company?  IAO:  - What did your research team do after making the decision?  - What kind of activities has IAO initiated to help researchers promote collaborative R&D projects?  - Did you encounter any difficulties in the collaborative R&D projects and how did you overcome these difficulties with the help of IAO?  - How did partnership management influence the development of collaborative R&D projects? |
|  | **Reconfiguring** | - Looking back at the whole process of the innovation project, what did your team (or your company) do differently compared to the past?  - From your point of view, what is the biggest contribution of IAO (or D-lab) in driving your innovation project?  - Along the way, has your team (or your company) changed anything strategically (i.e., absorbing new organizational structure, and business models)? Why and how?  - Regarding the collaborative partners, who are you separated from? Who are the existing or future partners at this stage? |
